# Elucidating spatial heterogeneities in 3D printed thermoplastic elastomers using micro-beam small-angle x-ray scattering

**DOI:** 10.1063/4.0000983

**Published:** 2025-10-27

**Authors:** Alice S Fergerson, Emily C Davidson

**Affiliations:** Princeton University, Princeton, NJ, USA

## Abstract

3D printing is uniquely capable of producing architectures with hierarchically ordered structure from the nano-scale through the macro-scale (cm). One primary approach by which 3D printing can tune nano- and meso-scale material anisotropy is through the application of shear and extensional flows to an intrinsically nanostructured ink, as these flows can induce orientation and anisotropic functional properties along the print path. In particular, we have studied the effects of varying the 3D printing-induced flow history on the nanostructural and mechanical anisotropy in cylinder-forming styrenic thermoplastic elastomers (TPEs), which can achieve nearly two orders of magnitude of mechanical anisotropy via 3DP. Our initial work demonstrated the importance of extensional flow in achieving a consistent and high degree of functional anisotropy. However, structural characterization using bench-top x-ray sources with a relatively large (>0.1 mm) beam footprint is unable to sufficiently capture the heterogeneity which can result from the spatially varied flow history applied during 3D printing. Here we leverage micro-focused x-ray capabilities at CHESS FMB and BNL 12-ID to elucidate the effects of these spatially heterogeneous flows on the nanostructural alignment and ordering within 3D printed TPE filaments. These synchrotron-based techniques are uniquely capable of providing insights into the local effects of a heterogeneous flow history, such as spatial variations in nanostructural alignment and, in select material systems, localized flow-induced transitions between block copolymer nanostructures.

